# First-Line Treatment Options for PD-L1–Negative Non-Small Cell Lung Cancer: A Bayesian Network Meta-Analysis

**DOI:** 10.3389/fonc.2021.657545

**Published:** 2021-06-23

**Authors:** Ling Peng, Wen-Hua Liang, De-Guang Mu, Song Xu, Shao-Dong Hong, Justin Stebbing, Fei Liang, Yang Xia

**Affiliations:** ^1^ Department of Respiratory Disease, Zhejiang Provincial People’s Hospital, Hangzhou, China; ^2^ National Clinical Research Center for Respiratory Disease, The First Affiliated Hospital of Guangzhou Medical University, Guangzhou, China; ^3^ Department of Lung Cancer Surgery, Lung Cancer Institute, Tianjin Medical University General Hospital, Tianjin, China; ^4^ Department of Medical Oncology, Sun Yat-sen University Cancer Center, Guangzhou, China; ^5^ Division of Cancer, Department of Surgery and Cancer, Imperial College London, London, United Kingdom; ^6^ Department of Biostatistics, Zhongshan Hospital, Fudan University, Shanghai, China; ^7^ Department of Respiratory and Critical Care Medicine, The Second Affiliated Hospital, School of Medicine, Zhejiang University, Key Laboratory of Respiratory Disease of Zhejiang Province, Hangzhou, China

**Keywords:** programmed death-ligand 1, non-small cell lung cancer, immune checkpoint inhibitor, network meta-analysis, immunotherapy

## Abstract

**Background:**

First-line treatment strategies for programmed death-ligand 1 (PD-L1) negative non-small cell lung cancer (NSCLC) patients include chemotherapy and combination with anti-angiogenesis drugs and/or immune checkpoint inhibitor. We conducted a Bayesian network meta-analysis to evaluate the efficacy of these therapeutic options.

**Methods:**

We included phase III randomized controlled trials comparing two or more treatments in the first-line setting for NSCLC, including data in PD-L1–negative patients. First-line strategies were compared and ranked based on the effectiveness in terms of overall survival (OS) and progression-free survival (PFS). A rank was assigned to each treatment after Markov Chain Monte Carlo analyses.

**Results:**

Fourteen trials involving 14 regimens matched our eligibility criteria. For OS, none of the treatment were significantly more effective than chemotherapy. Nivolumab plus ipilimumab plus chemotherapy was probably the best option based on analysis of the treatment ranking (probability = 30.1%). For PFS, nivolumab plus chemotherapy plus bevacizumab, atezolizumab plus chemotherapy plus bevacizumab, and atezolizumab plus chemotherapy were statistically superior to chemotherapy in pairwise comparison. Nivolumab plus chemotherapy plus bevacizumab was likely to be the preferred option based on the analysis of the treatment ranking (probability = 72.9%).

**Conclusions:**

Nivolumab plus chemotherapy, in combination with angiogenesis inhibition or anti-cytotoxic T-lymphocyte–associated antigen 4 (CTLA-4), had maximal benefits for NSCLC patient of PD-L1–negative expression. These findings may facilitate individualized treatment strategies. Safety at an individual patient level should be considered in decision making. Further validation is warranted.

## Introduction

Non-small cell lung cancer (NSCLC) accounts for ~85% of all lung cancer cases, and the prognosis for patients with advanced/metastatic NSCLC remains limited (1). Platinum-based chemotherapy has long been the first-line treatment of choice for advanced NSCLC patients who do not harbor activating driver mutations. Checkpoint blockade has led to a paradigm shift in the treatment landscape of NSCLC, making long-term survival possible (2).

Thus far, several effective first-line systemic treatment options have been shown to be effective in advanced NSCLC. Programmed death-ligand (PD-L1) expression on tumor or immune cells emerged as the first potential predictive biomarker for the sensitivity to immune checkpoint blockade and patient stratification (3). For NSCLC patients with PD-L1 expression in ≥50% of tumor cells, pembrolizumab confers a superior progression-free survival (PFS) and overall survival (OS) compared with platinum-doublet chemotherapy in the first-line setting (4). For PD-L1 expression of 1% to 49%, programmed death-1 (PD-1) or PD-L1 inhibition has been shown to be comparable to chemotherapy (5, 6). In contrast, for patients with negative PD-L1 expression, no definite optimal therapeutic strategy has been defined. Most importantly, this group accounts for about half of the whole NSCLC patient population (7). A lack of head-to-head randomized controlled trials (RCTs) comparing chemotherapy, anti-angiogenesis drugs, and immunotherapies leaves uncertainty regarding optimal first-line treatment for advanced NSCLC patients with negative PD-L1 expression.

Network meta-analysis offers the unique opportunity to perform indirect comparisons between treatments never directly compared in RCTs but compared to a common treatment, as well as to rank multiple treatments (8). The present study aims to probe optimal therapeutic management with advanced NSCLC with negative PD-L1 expression.

## Materials and Methods

### Search Strategy

A literature search was performed using databases including PubMed, Embase, and Cochrane databases. The upper date limit of October 30, 2020, was applied, with no lower date limit. Our search strategy included the following Medical Subject Headings (MeSH) terms and keywords: “NSCLC”, “(advanced) or (metastatic) or (stage IV)”, “(first-line) or (untreated) or (front-line)”. Searches were performed using the filter “clinical trial” or “study” or “investigation” or “phase 3”. We also reviewed abstracts and presentations from conference proceedings, including American Society of Clinical Oncology (ASCO), World Conference on Lung Cancer (WCLC), European Society for Medical Oncology (ESMO), European Lung Cancer Conference (ELCC), and American Association for Cancer Research (AACR). To ensure that no RCTs were missing, reference lists of published reviews, meta-analyses, and included RCTs were manually checked, and www.clinicaltrials.gov was searched.

### Study Selection

**Figure 1:**
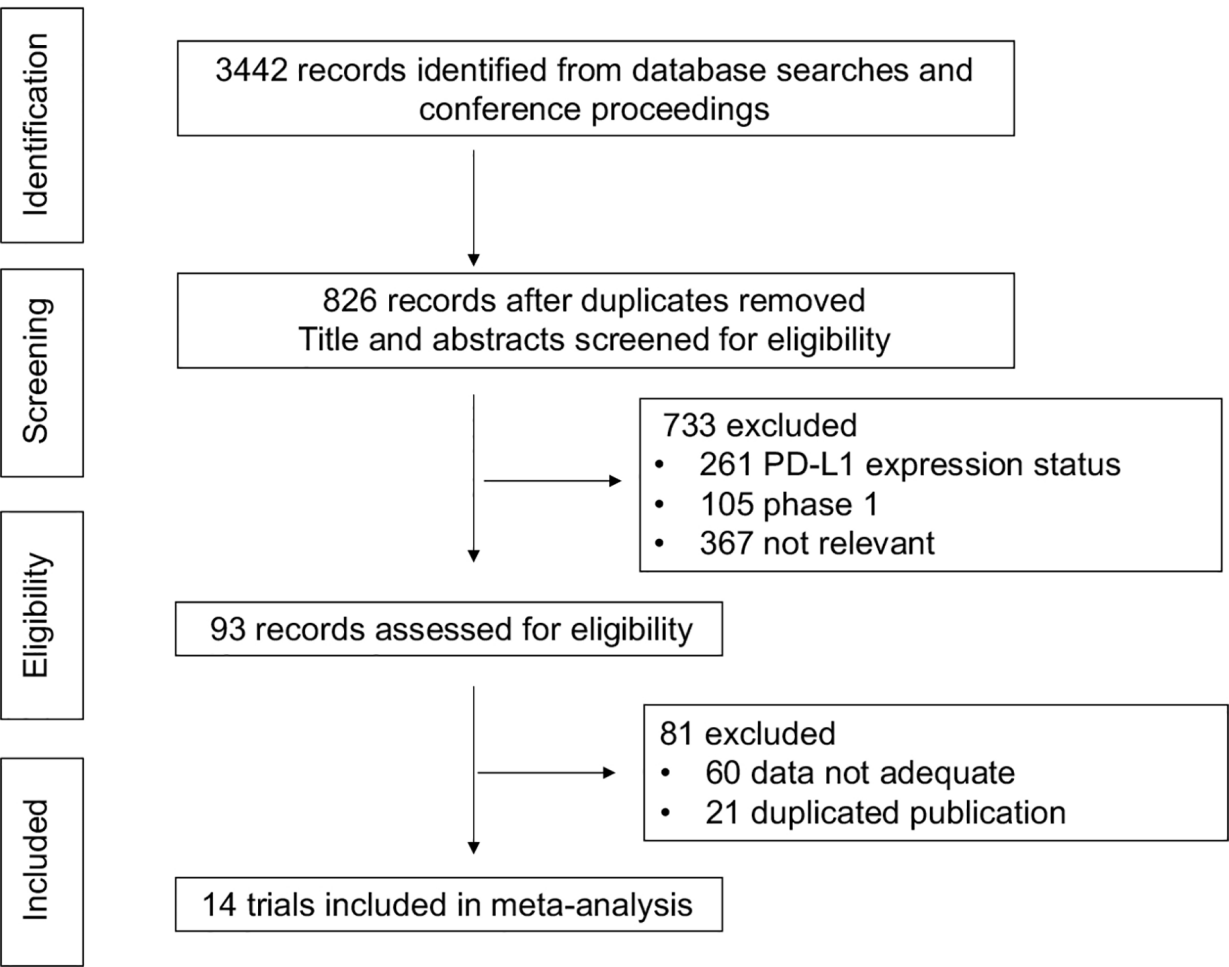
Selection process for the trials included in the meta-analysis. PRISMA diagram. NSCLC, non-small cell lung cancer; RCT, randomized-controlled trial.

Eligibility criteria for inclusion in this meta-analysis were as follows: (1) prospective phase III RCTs in patients with advanced NSCLC who had received no previous treatment for metastatic disease; (2) English language; (3) data available regarding PD-L1 expression negative population; and (4) in cases of duplicate publications, only the most recent and updated report of the clinical trial were also included. Review articles, non-randomized trials, and observational studies, non-English studies were excluded from the analysis. The selection process is shown in **Figure 1**.

Articles that could not be categorized based on title and abstract alone were retrieved for full-text review. Disagreements were resolved by consensus between the authors. To determine the issue of multiple publications from the same data sets, we confirmed clinical trial information, such as the trial number and the period of patient recruitment of the articles. We also assessed the eligibility of the articles and abstracts identified by the search, and discrepancies were resolved by consensus. Study quality was assessed using the Jadad five-item scale, which takes into account randomization, double blinding, and withdrawals. The final score ranged from 0 to 5 (9).

### Data Extraction

The meta-analysis was performed based on outcomes coming from the included studies. Data were extracted from eligible studies, which include the following items: study name, year of publication, source of publication, histology, number of patients, treatment arm and control arm, hazard ratio (HR), and 95% confidence intervals (CIs) of PFS and OS. In the case of trials that did not report PD-L1 expression subgroup, we reviewed each published trial’s supplementary material. If data from any of the above categories were not reported in the study, items were treated as NR (not reported). The primary variables of interest were HRs with 95% CIs for OS or PFS.

### Statistical Analysis

All calculations were performed using R (version 4.0.2) and STATA (version 14.0, Stata Corp LP, College Station, TX). OS and PFS were treated as time-to-event variables; therefore, these parameters were expressed as HR and 95% CI for each study. The primary endpoints of this network meta-analysis were the HRs for OS and PFS in PD-L1–negative patients. The Bayesian network meta-analysis (NMA) used a non-informative uniform prior to distribution to the parameters. For each outcome, three Markov chains with different starting values, generated using the method described by Gelman and Rubin were run in parallel for 100,000 iterations to obtain the posterior distribution. We used 10,000 burn-ins and a thinning interval of 10 for each chain. The model fit of each analysis was assessed by deviance information criterion (DIC) (10). Result heterogeneity across studies was evaluated with Cochrane’s Q statistic and quantified with the inconsistency statistic (*I^2^*). Statistical significance was considered at *p* less than 0.05, and heterogeneity was considered low, moderate, or high for *I^2^* values under 25%, between 25% and 50%, and over 50%, respectively (11). Effect sizes for the Bayesian network meta-analysis were described with 95% credible interval (CrL), the Bayesian equivalent of 95% CIs. Relative ranking of OS and PFS was presented as the probabilities. The probability of each regimen being the best among all regimens was computed by ranking the relative efficacies of all regimens in each iteration and then calculating the proportion of each regimen being ranked first across all iterations, which equals to 1 when a treatment is certain to be the best and 0 when a treatment is certain to be the worst.

## Results

### Study Selection and Characteristics

We found 4,125 potentially relevant articles. After initial exclusion of irrelevant, duplicate, and non-randomized studies, 14 original studies were considered eligible for the meta-analysis (**Figure 1**). The major baseline characteristics of the 14 eligible studies were represented in **Table 1**. Ten studies were double-arm design, whereas the remaining four referring three-arms. Overall, there were 14 different treatment strategies: chemotherapy, chemotherapy plus bevacizumab, atezolizumab plus chemotherapy, atezolizumab plus chemotherapy plus bevacizumab, nivolumab plus chemotherapy, nivolumab plus ipilimumab, nivolumab plus ipilimumab plus chemotherapy, nivolumab plus chemotherapy plus bevacizumab, pembrolizumab plus chemotherapy, caremlizumab plus chemotherapy, durvalumab, durvalumab plus tremelimumab, tislezumab plus chemotherapy, and sintilimab plus chemotherapy.

**Table 1:** Main characteristics and results of the eligible studies.

No	Year	Author	Source	Study	Histology	Phase	Sample size	Treatment	Control	OS (95% CI)	PFS (95% CI)
1	2019	West	Lancet Oncol	IMpower130 (12)	NSQ	3	356	Atezo+CT	CT	0.81 (0.61–1.08)	0.72 (0.56-0.91)
2	2020	Jotte	JTO	IMpower131 (13)	SQ	3	331	Atezo+CT	CT	0.87 (0.67–1.13)	0.81 (0.64–1.03)
3	2021	Papadimitrakopoulou	JTO	IMpower132 (14)	NSQ	3	143	Atezo+CT	CT	NR	0.45 (0.31–0.64)
4	2020	Socinski	AACR	IMpower150 (15)	NSQ+SQ	3	575	Atezo+CT	CT+Bev	0.96 (0.76–1.22)	1.10 (0.89–1.36)
								Atezo+CT+Bev	CT+Bev	0.90 (0.71–1.14)	0.77 (0.61–0.99)
5	2020	Zhou	Lancet Respir Med	CAMEL (16)	NSQ+SQ	3	118	Camre+CT	CT	NR	0.74 (0.44–1.23)
6	2020	Rizvi	JAMA Oncol	MYSTIC (17)	NSQ+SQ	3	254	Durva	CT	1.18 (0.86–1.62)	NR
								Durva+Treme	CT	0.73 (0.51–1.04)	NR
7	2020	Ramalingam	ASCO	CheckMate 227 Part 1b (18)	NSQ+SQ	3	550	Nivo+Ipi	CT	0.64 (0.51–0.81)	0.75 (0.59–0.95)
								Nivo+CT	CT	0.82 (0.66–1.03)	0.73 (0.58–0.92)
8	2021	Paz-Ares	Lancet Oncol	CheckMate 9LA (19)	NSQ+SQ	3	264	Nivo+Ipi+CT	CT	0.62 (0.45–0.85)	NR
9	2020	Lee	ESMO	ONO-4538-52/TASUKI-52 (20)	NSQ	3	550	Nivo+CT+Bev	CT+Bev	NR	0.55 (0.38–0.78)
10	2018	Gandhi	NEJM	KEYNOTE-189 (21)	NSQ	3	190	Pembro+CT	CT	0.59 (0.38–0.92)	0.75 (0.53–1.05)
11	2018	Paz-Ares	NEJM	KEYNOTE-407 (22)	SQ	3	104	Pembro+CT	CT	0.61 (0.38–0.98)	0.68 (0.47–0.98)
12	2020	Yang	JTO	ORIENT-11 (23)	NSQ	3	397	Sinti+CT	CT	NR	0.66 (0.41–1.09)
13	2021	Wang	JAMA Oncol	RATIONALE-307 (24)	SQ	3	91	Tisle+CT	CT	NR	0.64 (0.37–1.10)
										NR	0.69 (0.41–1.18)
14	2020	Lu	ESMO	RATIONALE-304 (25)	NSQ	3	334	Tisle+CT	CT	NR	0.76 (0.47–1.22)

Summary table of studies included in the meta-analysis.

OS, overall survival; PFS, progression-free survival; CI, confidence interval; CT, chemotherapy; Atezo, atezolizumab; Bev, bevacizumab; Nivo, nivolumab; Ipi, ipilimumab; Pembro, pembrolizumab; Carem, caremlizumab; Durva, durvalumab; Treme, tremelimumab; Tisle, tislezumab; Sinti, sintilimab; NR, not reported.

Studies were chosen and systemically reviewed in accordance with the Preferred Reporting Items for Systematic Reviews and Meta-Analyses (PRISMA) statement (26). Similarity was evaluated by reviewing characteristics of the trials with respect to any of those characteristics that are potential treatment effect modifiers, assuring validity of making indirect comparisons. It was impossible to calculate the Jadad’s score for 2 of the studies (RATIONALE-304 and ONO-4538-52), which have not yet been published at the time of the analysis. The Jadad’s score was evaluated for the rest 12 studies with scores ranging from 3 to 5.

### Network Meta-Analysis of OS

**Figure 2:**
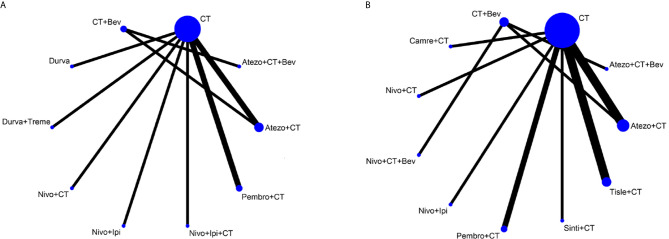
Network plot for effectiveness of 10 and 9 different treatment modalities for patients with PD-L1–negative expression for OS **(A)** and PFS **(B)**, respectively. Circles represent the intervention as a node in the network and their size is proportional to the number of included studies; lines represent direct comparisons within the frame of randomized clinical trials (RCTs); the line thickness indicates the number of RCTs included in each comparison. CT, chemotherapy; Atezo, atezolizumab; Bev, bevacizumab; Nivo, nivolumab; Ipi, ipilimumab, Pembro, pembrolizumab; Carem, caremlizumab; Durva, durvalumab; Treme, tremelimumab; Tisle, tislezumab; Sinti, sintilimab; NR, not reported.

**Figure 3:**
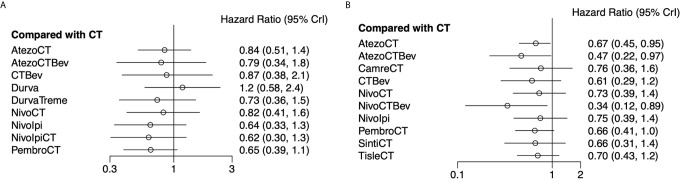
Forest plots showing OS **(A)** and PFS **(B)** hazard ratio analyses. Efficacy of 10 and 11 treatment modalities for OS and PFS, respectively. Outcome measure: hazard ratio (HR). PrI, predictive interval; CT, chemotherapy; Atezo, atezolizumab; Bev, bevacizumab; Nivo, nivolumab; Ipi, ipilimumab; Pembro, pembrolizumab; Carem, caremlizumab; Durva, durvalumab; Treme, tremelimumab; Tisle, tislezumab; Sinti, sintilimab; NR, not reported.

**Figure 4:**
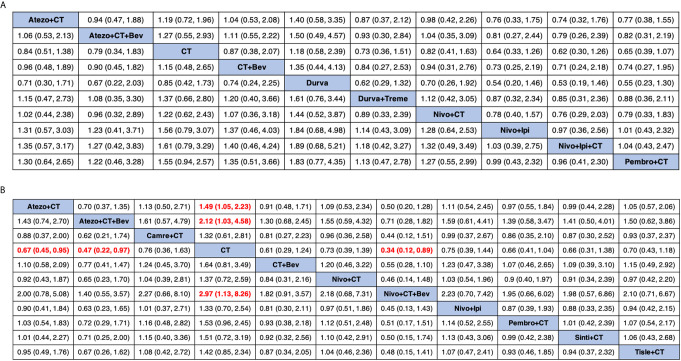
Comparative efficacy of treatments for OS **(A)** and PFS **(B)** in network meta-analysis. Comparisons should be read from left to right. HR (95% credible interval) for comparisons is in cells in common between column-defining and row-defining treatment. Bold cells are significant. HR >1 favors row-defining treatment, and HR <1 favors column-defining treatment. CT, chemotherapy; Atezo, atezolizumab; Bev, bevacizumab; Nivo, nivolumab; Ipi, ipilimumab; Pembro, pembrolizumab; Carem, caremlizumab; Durva, durvalumab; Treme, tremelimumab; Tisle, tislezumab; Sinti, sintilimab; NR, not reported.

**Figure 5:**
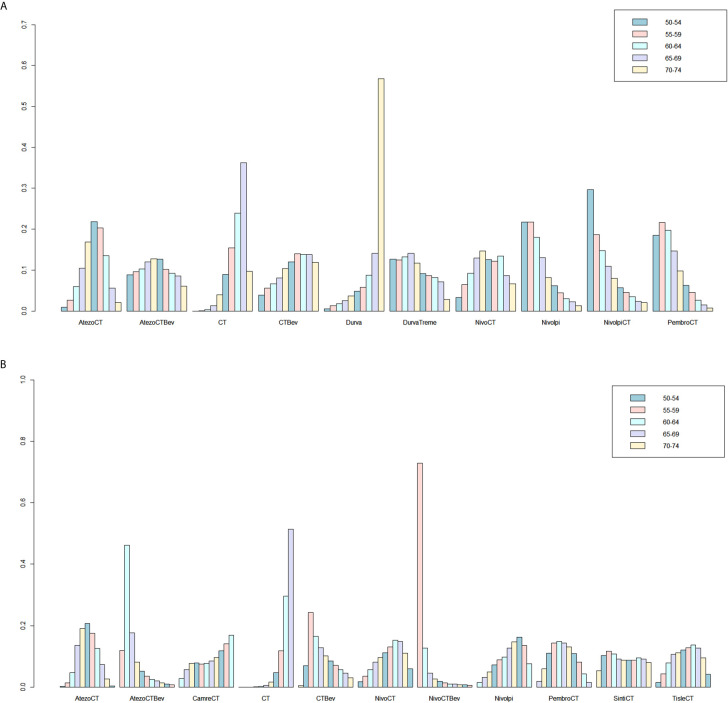
Ranking probabilities based on the multiple comparisons on OS **(A)** and PFS **(B)** in NSCLC patients with PD-L1–negative expression. CT, chemotherapy; Atezo, atezolizumab; Bev, bevacizumab; Nivo, nivolumab; Ipi, ipilimumab; Pembro, pembrolizumab; Carem, caremlizumab; Durva, durvalumab; Treme, tremelimumab; Tisle, tislezumab; Sinti, sintilimab; NR, not reported.

Eight studies provided HR values for OS. The comparisons between treatments were shown by network plot (**Figure 2**). The forest plot of OS for pairwise comparison results were presented in **Figure 3**. In pairwise comparison, compared with chemotherapy, none of the treatments had a significant lower hazard risk of OS. The results providing indirect comparisons between treatments are presented in **Figure 4**, with none of the treatments performing significantly better than other treatment regimen in terms of OS. Comparative efficacy of treatments for OS based on treatment ranking was shown in **Figure 5** and **Table 2**, among which, combination of nivolumab and ipilimumab and chemotherapy was the most possible therapy to be ranked as first for OS (probability = 30.1%), nivolumab plus ipilimumab ranked the second (probability = 22.4%), and pembrolizumab plus chemotherapy ranked the third (probability = 18.8%). Comparing the DIC between the consistency and inconsistency models suggests that the consistency model has a similar fit to the data with inconsistency model (21.35 *vs.* 21.39). The overall heterogeneity assessment of the results showed that the heterogeneity was low for OS (*I^2^* = 0%).

**Table 2:** Ranking probabilities of different first-line treatment strategies for PD-L1–negative NSCLC patients.

	Ranking probability for the best (OS, %)	Ranking probability for the best (PFS, %)
Atezo + CT	0.71	0.20
Atezo + CT + Bev	8.46	11.93
Camre + CT	NR	2.71
CT	0.00	0.00
CT + Bev	3.46	0.46
Durva	0.48	NR
Durva + Treme	12.61	NR
Nivo + CT	2.97	1.77
Nivo + CT + Bev	NR	**72.92**
Nivo + Ipi	22.39	1.44
Nivo + Ipi + CT	**30.09**	NR
Pembro+CT	18.81	1.86
Sinti + CT	NR	5.26
Tisle + CT	NR	1.45

OS, overall survival; PFS, progression-free survival; CT, chemotherapy; Atezo, atezolizumab; Bev, bevacizumab; Nivo, nivolumab; Ipi, ipilimumab; Pembro, pembrolizumab; Carem, caremlizumab; Durva, durvalumab; Treme, tremelimumab; Tisle, tislezumab; Sinti, sintilimab; NR, not reported. Bold means the the highest ranking probablity

### Network Meta-Analysis of PFS

As for PFS, there were nine studies reported the HR values (**Figure 2**). As shown in **Figure 3**, nivolumab plus chemotherapy plus bevacizumab (HR, 0.34; 95% CrI, 0.12–0.89), atezolizumab plus chemotherapy plus bevacizumab (HR, 0.47; 95% CrI, 0.22–0.97), and atezolizumab plus chemotherapy (HR, 0.67; 95% CrI, 0.45–0.95) were statistically superior to chemotherapy in pairwise comparison. Indirect comparison results were illustrated in **Figure 4**, with nivolumab plus chemotherapy plus bevacizumab, atezolizumab plus chemotherapy plus bevacizumab, and atezolizumab plus chemotherapy have better PFS than chemotherapy. The probabilities of rank plot (**Figure 5** and **Table 2**) were as follows: combination of nivolumab, chemotherapy, and bevacizumab was most likely to be the best regimen (probability = 72.9%), atezolizumab plus chemotherapy plus bevacizumab ranked the second (probability = 11.9%). The DIC between the consistency and inconsistency models suggests that the consistency model has a similar fit to the data inconsistency model (27.05 *vs.* 27.06). The overall heterogeneity assessment of the results showed that the heterogeneity was low for PFS (*I^2^* = 22.1%).

## Discussion

The PD-L1 axis is regulated by different stimuli through multiple levels, including genomic, transcriptional, post-transcriptional, translational, and post-translational levels (27). PD-L1 expression has been proposed as distinct biomarker of response to PD-(L)1 inhibitor. In NSCLC, PD-L1 expression is highly variable and is associated with distinct clinicopathologic and genomic features (28). Clinical studies in NSCLC have demonstrated that PD-L1 expression on tumor and/or immune cells has a positive correlation with the efficacy of anti-PD-(L)1 therapy. A real-world EXPRESS study evaluated the PD-L1 expression profile in locally advanced or metastatic NSCLC, revealing that PD-L1–negative patients account for about 40% to 53% (7). Efficacies of PD-(L)1 blockade treatment in patients that are PD-L1 positive or negative are significantly different (29). Here, our analysis is designed to answer the open question of the optimal therapeutic management in advanced NSCLC with negative PD-L1 expression.

The expression of PD-L1 can be classified into constitutive and inducible expression depending on the extrinsic or intrinsic stimuli (30). Constitutive expression is dependent on cell genomics, while inducible PD-L1 expression is dependent on exposure of cells to cytokines, such as IFNγ, TNFα, IL-1α, and IL-1β *via* TLRs or IFN receptors (31). PD-L1–negative expression of a tumor is sometimes considered as the tumor being “cold” to use a somewhat colloquial term (32). The absence of PD-L1 expression on tumor cells might, for example, indicate impaired IFN-γsignaling (33). By turning “cold” tumors to “hot”, combination strategies emerge, which involve different immune checkpoint inhibitors (ICIs) with chemotherapy, anti-angiogenesis, and other new classes drugs or, for example, oncolytic viruses (34).

The 14 treatment modalities in our meta-analysis for PD-L1–negative NSCLC can be categorized into seven types: chemotherapy, chemotherapy plus angiogenesis inhibition, mono anti-PD-(L)1, anti-PD-(L)1 plus chemotherapy, anti-PD-(L)1 plus anti-cytotoxic T-lymphocyte–associated antigen 4 (CTLA-4), anti-PD-(L)1 plus anti-CTLA-4 plus angiogenesis inhibition, and anti-PD-(L)1 plus anti-CTLA-4 plus chemotherapy.

Chemotherapy was previously considered to be immunosuppressive, whereas cytotoxic drugs may also exert an immunomodulatory role in NSCLC and other solid tumors (35). A recent pooled analysis of three randomized trials assessing PD-L1–negative patients receiving pembrolizumab with chemotherapy combination strategy confirmed a clinically meaningful benefit improvement (36). The inclusion of HR from phase II studies might influence the results; therefore, only phase III trials were included in this analysis, leaving phase II KEYNOTE-021G trial (37) ineligible for our analysis.

The rationale for combining anti-angiogenesis drug with ICIs rests in aspects, including immuno-metabolism and tumor microenvironment (38), which leads to a synergistic effect. Therapeutic regimens of chemotherapy with anti-angiogenesis drugs, such as ECOG-4599 (39), BEYOND (40), were not included in the network meta-analysis because of lack of PD-L1 expression status. In the IMpower150 trial, ACP (atezolizumab plus chemotherapy) and BCP (bevacizumab plus chemotherapy) had similar outcomes for the PD-L1–negative population (15).

Another combination choice for PD-(L)1 inhibitor is the combination of a CTLA-4 inhibitor, as used by CheckMate 227. Anti-PD-1 and anti–CTLA-4 dual blockade offers a “chemo-free” choice for PD-L1–negative patients. Dual blockade of CTLA-4 and PD-1 therapy is sufficient to induce unique cellular responses compared with either monotherapy, which has been proven in preclinical studies (41). However, the toxicity of adding another ICI to a PD-(L)1 inhibitor leads to more toxicity (42). In our network meta-analysis, we have no data for toxicity regarding PD-L1–negative patients receiving different treatment strategies. However, based on a previous meta-analysis, combination with CTLA-4 inhibitor might lead to more toxicities (42).

For OS and PFS, based on treatment ranking probabilities, nivolumab plus chemotherapy plus ipilimumab/bevacizumab ranked first, respectively. However, nivolumab plus chemotherapy plus ipilimumab (CheckMate 9LA) did not report PFS subgroup data regarding PD-L1–negative patients, whereas nivolumab plus chemotherapy plus bevacizumab (ONO-4538-52) did not report OS data in PD-L1 negative patients. These subgroup data are missing and will thus impact the result of network meta-analysis comparison. Although these four-drug combinations prevailed in survival than the other regimens by ranking probability, more toxicities might also occur in four-drug combinations. In CheckMate 9LA trial, three times of treatment-related adverse events (TRAEs) of nivolumab plus ipilimumab plus two cycles of chemotherapy than control arm render a four-drug combination, an option for PD-L1 negative patients but may not be the standard of care.

Our meta-analysis has several limitations. First, there were no clinical trials investigating only PD-L1–negative NSCLC patients. Therefore, data were derived from subgroup analysis of each primary study, and none of these trials were powered to detect the difference in OS or PFS in the PD-L1–negative subgroup, which explained why none of the treatment were significantly more effective in OS than chemotherapy. Some of the trials did not report OS, making comparisons not identical between PFS and OS. Second, the antibodies using to detect PD-L1 expression varied in different trials. Spatial and temporal heterogeneity of PD-L1 expression and different test platforms have made PD-L1 an imperfect biomarker. However, PD-L1 expression especially in tumor cells is currently the most widely used biomarker in patient stratification. Third, we have no access to toxicity data for patients with PD-L1–negative expression, and such expression is often heterogenous (43). Balancing the benefit/risk to a specific patient population is always challenging (44).

In summary, our meta-analysis is the first study to systematically investigate the treatment options for PD-L1–negative patients of NSCLC. In the absence of an RCT directly comparing first-line treatment options for NSCLC of PD-L1–negative expression, our findings suggest that two combined therapies, nivolumab plus ipilimumab plus chemotherapy, and nivolumab plus chemotherapy plus bevacizumab, both appear the most effective therapeutic strategies for this patient population in terms of OS and PFS, respectively. Further research, particularly phase III RCTs comparing treatment options in PD-L1–negative patients are required.

## Data Availability Statement

The original contributions presented in the study are included in the article/supplementary material. Further inquiries can be directed to the corresponding authors.

## Author Contributions

YX and FL had full access to all the data in the study and takes responsibility for the integrity of the data and the accuracy of the data analysis. YX, WHL, and LP participated in the concept and design. All authors participated in the acquisition, analysis, or interpretation of data. All authors participated in the drafting of the manuscript. All authors participated in the critical revision of the manuscript. LP, FL, and YX participated as the administrative, technical, or material support. YX and FL participated in the supervision. All authors contributed to the article and approved the submitted version.

## Funding

This study was partially supported by Natural Science Foundation of Zhejiang Province, China (grant LY19H160041). The funders had no role in study design, data collection and analysis, decision to publish, or preparation of the manuscript.

## Conflict of Interest

JS, the Editor-in-Chief of Oncogene has sat on SABs for Vaccitech, Heat Biologics, Eli Lilly, Alveo Technologies, Pear Bio, Agenus, Equilibre Biopharmaceuticals, Graviton Bioscience Corporation, Celltrion, Volvox, Certis Oncology Solutions, Greenmantle, Zedsen, Bryologyx and Benevolent AI. He has consulted with Lansdowne partners and Vitruvian. He sits on the Board of Directors for Xerion and BB Biotech Healthcare Trust PLC.

The remaining authors declare that the research was conducted in the absence of any commercial or financial relationships that could be construed as a potential conflict of interest.
